# Contributions of Red Blood Cell Sedimentation in a Driving Syringe to Blood Flow in Capillary Channels

**DOI:** 10.3390/mi13060909

**Published:** 2022-06-08

**Authors:** Yang Jun Kang

**Affiliations:** Department of Mechanical Engineering, Chosun University, 309 Pilmun-daero, Dong-gu, Gwangju 61452, Korea; yjkang2011@chosun.ac.kr; Tel.: +82-62-230-7052; Fax: +82-62-230-7055

**Keywords:** red blood cell sedimentation, blood viscosity, blood junction pressure, microfluidic device, empirical formula, hematocrit, blood flow imaging, biophysical property

## Abstract

The erythrocyte sedimentation rate (ESR), which has been commonly used to detect physiological and pathological diseases in clinical settings, has been quantified using an interface in a vertical tube. However, previous methods do not provide biophysical information on blood during the ESR test. Therefore, it is necessary to quantify the individual contributions in terms of viscosity and pressure. In this study, to quantify RBC sedimentation, the image intensity (*I_b_*) and interface (*β*) were obtained by analyzing the blood flow in the microfluidic channels. Based on threshold image intensity, the corresponding interfaces of RBCs (*I_b_* > 0.15) and diluent (*I_b_* < 0.15) were employed to obtain the viscosities (*µ_b_*, *µ*_0_) and junction pressures (*P_b_*, *P*_0_). Two coefficients (*CH*_1_, *CH*_2_) obtained from the empirical formulas (*µ_b_ = µ*_0_ [1 + *CH*_1_], *P_b_ = P*_0_ [1 + *CH*_2_]) were calculated to quantify RBC sedimentation. The present method was then adopted to detect differences in RBC sedimentation for various suspended blood samples (healthy RBCs suspended in dextran solutions or plasma). Based on the experimental results, four parameters (*µ*_0_, *P*_0_, *CH*_1_, and *CH*_2_) are considered to be effective for quantifying the contributions of the hematocrit and diluent. Two coefficients exhibited more consistent trends than the conventional ESR method. In conclusion, the proposed method can effectively detect RBC sedimentation.

## 1. Introduction

The biophysical properties of blood (i.e., viscosity, red blood cell (RBC) deformability, RBC aggregation [1,2,3,4], and erythrocyte sedimentation rate (ESR) [5,6,7]) provide vital information on the progress or severity of hemorheological disorders and diseases (e.g., diabetes [8], sickle cell anemia [9], and malaria [10,11]) [12]. Among these, ESR, as a nonspecific parameter, has been commonly used to detect physiological and pathological diseases in clinical settings. the variation in ESR has been influenced by several factors, including plasma proteins, RBC membrane structures (i.e., morphology and surface-volume ratio), membrane elasticity, and hematocrit [13]. To shorten the test time of RBC sedimentation, it is necessary to decrease the hematocrit (Hct) of blood. Whole blood is then diluted with sodium citrate (or saline) at a ratio of 4:1. A vertical tube (inner diameter = 2.55 mm, length = 200 mm) is filled with diluted blood [14]. After an elapse of 1 h, the ESR is then calculated by detecting the interface between RBCs and the diluent in the vertical tube (i.e., ESR = RBC sedimentation for 1 h). Currently, the conventional method has been widely used because its facility and working principle are extremely simple. However, it requires a long measurement time (~1 h) and large blood consumption (~1 mL).

According to previous studies, Lee et al. have demonstrated a modified Westergren ESR method that adopted a microfluidic device for detecting malaria [11] or diabetes [15,16]. Yeom et al. have introduced an air-compression-release mechanism for accelerating ESR in a vertical syringe tube. A previous method has been employed to detect differences in the ESR between control blood and periodontitis blood [5]. Seelamantula et al. have suggested a hybrid imaging technique for computing the setting velocity of an individual RBC in a vertical microfluidic channel [17]. Elbuken et al. have proposed a portable microfluidic opto-electro-mechanical system [18]. A solenoid pinch valve has been used to disaggregate the RBCs. The light transmitted through the cartridge is measured using a photodetector. Because RBC sedimentation occurs in the vertical tube, the hematocrit increases in the lowest regions over time. Blood is supplied from the lowest regions of the tube to the microfluidic channel. Based on the strong relationship between hematocrit and microscopic image intensity of blood flow [19], Kang et al. has quantified variations in hematocrit in terms of the image intensity of blood flow with a specific microfluidic channel [20,21]. According to the previous methods, the ESR has been quantified by analyzing image intensity of blood flow. However, the image intensity of the blood flow does not provide quantitative or biophysical information on the mechanical properties related to blood flow. As RBC sedimentation is influenced simultaneously by RBCs and the diluent, it is necessary to quantify their individual contributions in terms of biophysical properties (i.e., viscosity [22], pressure [23,24]).

In this study, both contributions of RBCs and diluents during RBC sedimentation in a driving syringe are quantified in terms of viscosity and blood junction pressure. To demonstrate the proposed method, blood is supplied to a microfluidic device from the driving blood syringe. During blood delivery, the diluent is separated from the blood after an elapse of a certain time. Blood and diluent are then supplied sequentially to a microfluidic device. Using a microfluidic device, blood viscosity and blood junction pressure are quantified by monitoring the blood stream in the coflowing channel.

Compared to previous methods, the proposed method has distinctive advantages. First, it provides temporal variations in viscosity and pressure during RBC sedimentation in a driving syringe. Second, it suggests two coefficients (*CH*_1_, *CH*_2_) that represent the contributions of RBCs (hematocrit) to blood viscosity and pressure. Using two coefficients, it is possible to separate the individual effects of RBCs and diluents from RBC sedimentation in the driving syringe.

## 2. Materials and Methods

### 2.1. Microfluidic Device and Experimental Setup

**Figure 1:**
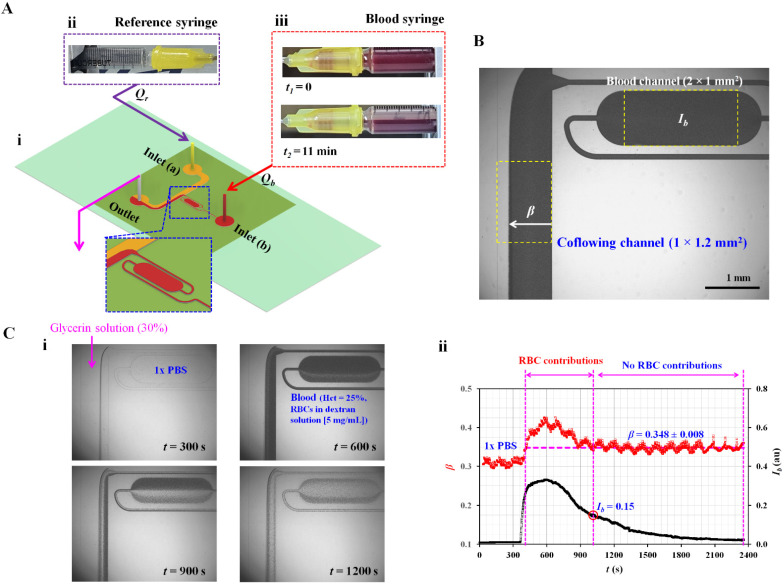
Proposed method for quantifying RBC sedimentation in blood syringes. (**A**) Schematic diagram of proposed method, which consisted of a microfluidic device and two syringe pumps. (**A**-**i**) Microfluidic device with blood channel, coflowing channel, two inlets (a and b), and one outlet. Each syringe pump was set to *Q_r_* (reference fluid) and *Q_b_* (blood) constantly over time. (**A**-**ii**) Snapshot for reference syringe. (**A**-**iii**) Two snapshots of the blood syringe captured at two specific times (i.e., *t*_1_ = 0 and *t*_2_ = 11 min). (**B**) Two parameters (*I_b_* and *β*) obtained by analyzing blood flow. Image intensity (*I_b_*) was quantified by analyzing blood flow selected within the blood channel with ROI (2 × 1 mm^2^). Blood-filled width (*β*) in the coflowing channel with ROI (1 × 1.2 mm^2^) was obtained by analyzing the interface. (**C**) Contributions of RBCs to blood flow in terms of the two parameters (*I_b_* and *β*). (**C**-**i**) Microscopic images showing blood flow in the blood channel and interface in the coflowing channel with an elapse of time (*t*) (*t* = 300, 600, 900, and 1200 s). (**C**-**ii**) Temporal variations in *β* and *I_b_*. When image intensity was less than 0.15 (*I_b_* < 0.15), the *β* remained as *β* = 0.348 ± 0.008.

Figure 1(A-i) shows a microfluidic device consisting of a coflowing channel, a blood channel, two inlets (a, b), and an outlet. The coflowing channel and blood channel had a rectangular channel with a low aspect ratio (width (*w*) = 1000 µm, depth (*h*) = 50 µm). Using microelectromechanical system fabrication techniques [25], a polydimethylsiloxane (PDMS, Sylgard 184, Dow Corning, Midland, MI, USA) slab was replicated from a four-inch silicon master mold. Two inlets (a, b) and one outlet were punched using a biopsy punch (outer diameter = 0.75 mm). The PDMS slab and glass slide were treated with oxygen plasma (CUTE-MPR, Femto Science Co., Hwaseong-si, Korea). Thereafter, both surfaces were strongly bonded after heat treatment condition (i.e., 100 °C for 10 min).

The microfluidic device was positioned on an inverted microscope (IX53, Olympus, Tokyo, Japan) with a 4× objective lens (NA = 0.1). Two kinds of polyethylene tubing (length = 400 mm, inner diameter = 250 µm) were tightly connected to two inlets (a, b). Additionally, one kind of polyethylene tubing (length = 200 mm, inner diameter = 250 µm) was fitted to the outlet. To remove air inside the channels and avoid nonspecific bonding of plasma proteins, bovine serum albumin (BSA, C_BSA_ = 1 mg/mL) was injected into the tubing connected to the outlet. The microfluidic channels filled with BSA were maintained for 10 min. Thereafter, the microfluidic channels were again filled with 1× PBS (phosphate-buffered saline) by injecting it through the outlet. As shown in Figure 1(A-ii), a syringe filled with reference fluid (30% glycerin solution) was installed into a syringe pump (neMESYS, Cetoni GmbH, Korbußen, Germany). The flow rate was set to 0.5 mL/h (*Q_r_* = 0.5 mL/h). Simultaneously, a syringe filled with blood was installed in the syringe pump. As shown in Figure 1(A-iii), the syringe pump was installed horizontally. As a preliminary demonstration, blood (Hct = 25%) was prepared by adding normal RBCs to a dextran solution (5 mg/mL). The flow rate of the blood syringe was set to 0.5 mL/h (*Q_b_* = 0.5 mL/h). From two snapshots captured at *t*_1_ = 0 and *t*_2_ = 11 min, RBC sedimentation occurred in the blood syringe. RBC sedimentation caused a decrease in hematocrit in the blood flowing in the microfluidic channels. Based on the two mechanical properties (i.e., blood viscosity and junction pressure) that are influenced by the hematocrit of blood, variations in RBC sedimentation were monitored over time.

### 2.2. Microscopic Imaging Acquisition and Processing for Image Intensity and Interface

A high-speed camera (FASTCAM MINI, Photron, Tokyo, Japan) was used to capture microscopic images for quantifying the image intensity as well as the interface. The camera was set to 2000 frames per second. Two microscopic images were captured sequentially at an interval of 1 s during each experiment. All experiments were conducted at a constant temperature of 20 °C.

To quantify RBC sedimentation in the blood syringe, two representative parameters (image intensity and interface) were quantified by analyzing microscopic images captured over time. As shown in Figure 1B, a specific region-of-interest (ROI, 2 × 1 mm^2^) was selected within the blood channel for quantifying the image intensity of the blood flow in the microfluidic channel. The image intensity of the blood flow (*I_b_*) was obtained by averaging the image intensities distributed over the ROI. Second, the interface between the blood stream and reference stream was detected within a specific ROI of the coflowing channel (1 × 1.2 mm^2^). Here, the grayscale image was converted into a binary image using a level-thresholding algorithm [26]. The width of the bloodstream (*w_b_*) was obtained by averaging the interfaces distributed over the ROI. The normalized interface (*β*) was calculated as *β* = *w_b_*/*w* by dividing the blood-filled width by channel width.

Blood and reference fluids were supplied to the microfluidic channel using two syringe pumps. Figure 1(C-i) shows microscopic images captured at specific times (*t*) (*t* = 300, 600, 900, and 1200 s). Initially, the blood channel and tubing connected to inlet (b) were filled with 1× PBS. By turning on the blood syringe pump, 1× PBS flowed in the blood channel and coflowing channel (*t* = 300 s). Subsequently, blood flowed in the blood channel and coflowing channel (*t* = 600 s). The RBC sedimentation caused a decrease in hematocrit (or image intensity) in the blood channel and coflowing channel (*t* = 900 s). At a specific time of *t* = 1200 s, the hematocrit of the blood flowing in the blood channel and coflowing channel decreased substantially. Figure 1(C-ii) shows the temporal variations in *β* and *I_b_*. When the blood flowed in both channels, *I_b_* and *β* increased substantially. Simultaneously, the RBC sedimentation resulted in decreasing *I_b_* over time. However, *β* remained unchanged after a certain period of time. Specifically, *β* remained as *β* = 0.348 ± 0.008 when the image intensity was less than 0.15 (i.e., *I_b_* < 0.15). From the preliminary investigation, RBCs in blood flow (i.e., hematocrit) had a strong influence on the variation in *β*, where the image intensity was greater than 0.15.

### 2.3. Blood Preparation

One bag of RBCs (~320 mL) was purchased from the Gwangju–Chonnam Blood Bank (Gwangju, South Korea). Each blood sample was adjusted to a specific hematocrit ranging from 30% to 50% by adding RBCs into specific dextran solutions or 1× PBS. Five kinds of dextran solutions (C_dex_ = 5, 7.5, 10, 15, and 20 mg/mL) were prepared by dissolving dextran powder (Leuconostoc spp., MW = 450–650 kDa, Sigma-Aldrich, St. Louis, MO, USA) in 1× PBS.

## 3. Results and Discussion

### 3.1. Validation of Junction Pressure and Estimation of Correction Factor

**Figure 2:**
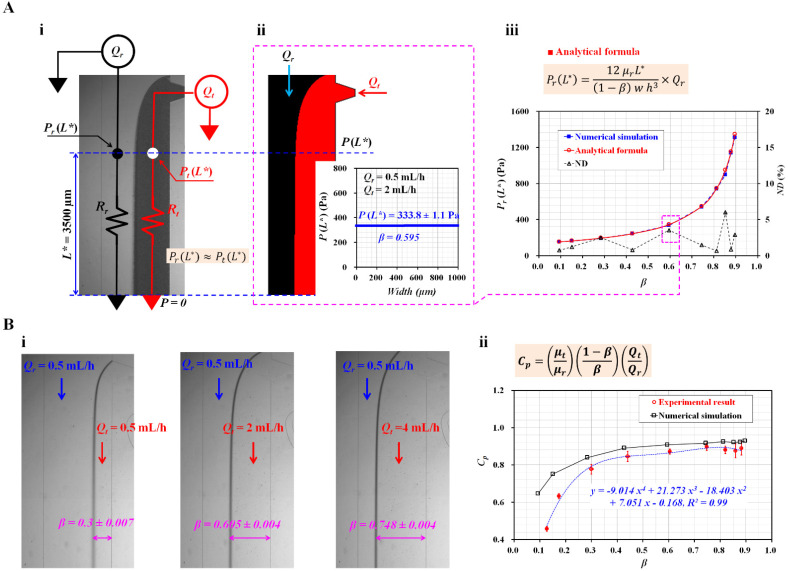
Validation of junction pressure and correction factor in the coflowing channel. (**A**) Comparison of junction pressures obtained using numerical simulation and analytical formula. (**A**-**i**) Discrete fluidic circuit model of the two fluids flowing in the coflowing channel. (**A**-**ii**) Pressure estimation through numerical simulation. The junction pressure and interface obtained were *P*(L* = 3500 µm) = 333.8 ± 1.1 Pa and *β* = 0.595. (**A**-**iii**) Variations in *P_r_* (L*) obtained using numerical simulation and analytical formula with respect to *β*. (**B**) Calculation of correction factor (*C_P_*) with respect to the interface. (**B**-**i**) Microscopic images showing variations in the interface with respect to the flow rate of test fluid (*Q_t_*) (*Q_t_* = 0.5, 2, and 4 mL/h). (**B**-**ii**) Variations in *C_P_* obtained from numerical simulations and experiments with respect to *β*. Based on the variations in *β* obtained from the experiments, the regression analysis resulted in a correction factor *C_p_* = −9.014*β*^4^ + 21.273*β*^3^ − 18.403*β*^2^ + 7.051*β* − 0.168 (*R*^2^ = 0.99).

To quantify the contribution of RBCs to blood flow, it was necessary to obtain two mechanical properties (i.e., blood viscosity and junction pressure). Here, both blood properties were obtained by analyzing the interface in the coflowing channel. Figure 2(A-i) shows the discrete fluidic circuit model of the two fluids partially flowing in the coflowing channel. The coflowing channel was represented by two fluidic resistances (*R_r_*, *R_t_*) connected in parallel. The flow rates of the reference and test fluids were denoted as *Q_r_* and *Q_t_*, respectively. The junction pressure at a distance (L* = 3500 µm) from the outlet (or the ground) was denoted as *P_r_* (L*) for the reference fluid stream and *P_t_* (L*) for the test fluid stream. According to a previous study [27], the analytical expression for each junction pressure is given as:

(1) $$P_{r}\left(L^{*}\right)=\left(\frac{12\;\mu_{r}\;L^{*}\;}{\left(1-\beta \right)\;w\;h^{3}}\right)\times Q_{r},$$

(2) $$P_{t}\left(L^{*}\right)=\left(\frac{12\;\mu_{t}\;L^{*}\;}{C_{p}\;\beta \;w\;h^{3}}\right)\times Q_{t}$$

In Equation (2), the correction factor (*C_P_*) was used to compensate for the difference between the real physical boundary and the mathematical modeling boundary. *µ_r_* and *µ_t_* denoted viscosities of reference fluid and test fluid, respectively. Additionally, w represented the channel width of the coflowing channel. As the coflowing channel had straight and rectangular channels, both streams had the same pressure (i.e., *P_r_* (L*) ≈ *P_t_* (L*)). Based on Equations (1) and (2), the correction factor (*C_P_*) was derived as:

(3) $$C_{P}=\left(\frac{\mu_{t}}{\mu_{r}}\right)\times \left(\frac{1-\beta }{\beta }\right)\times \left(\frac{Q_{t}}{Q_{r}}\right)$$

To validate the analytical expressions for the junction pressure (i.e., Equations (1) and (2)), numerical simulations were conducted using a commercial software (CFD-ACE+, Ver. 2021, ESI Group, Paris, France). The test and reference fluids were selected as 1× PBS and 30% glycerin solutions, respectively. The corresponding viscosities of each fluid were assumed to be *µ_r_* = 3 cP (reference fluid) and *µ_t_* = 1 cP (test fluid) [28]. The flow rate of each fluid was set to *Q_r_* = 0.5 mL/h and *Q_t_* = 2 mL/h. Figure 2(A-ii) shows the interface in the coflowing channel. The right panel shows the variations in *P*(L*) along the channel width. As expected, the junction pressure remained unchanged along the channel width. Both the streams had the same junction pressure at a specific location. The junction pressure and interface were obtained as *P*(L*) = 333.8 ± 1.1 Pa and *β* = 0.595, respectively. For convenience, the junction pressure without a correction factor (*C_P_*) (i.e., Equation (1)) was employed to validate the analytical expression in the coflowing channel. Figure 2(A-iii) shows the variations in *P_r_* (L*) obtained by the numerical simulation and the analytical formula with respect to *β*. The normalized difference (ND, Δ) was obtained by comparing the junction pressures obtained by the numerical simulation (■) and the analytical formula (○). The results indicate that the junction pressure increased significantly when the interface moved from the left wall (*β* = 0) to the right wall (*β* = 1). It increased significantly from 154 to 1346 Pa. In addition, ND was less than 6% when the interface was located from *β* = 0.1 to *β* = 0.9. From the results, Equation (1) could be used to obtain the junction pressure in the coflowing channel consistently.

To obtain blood viscosity in the coflowing channel, it was necessary to obtain the correction factor (*C_P_*) with the experimental approach. The flow rate of the reference fluid was set at 0.5 mL/h. According to a previous study [29], the correction factor was influenced by the sectional dimensions of the rectangular channel (i.e., width, depth), and the reference fluid. Thus, it was necessary to recalculate the correction factor. Here, the microfluidic channel was newly designed, and a 30% glycerin solution was selected as the reference fluid. PBS (1×) was used as the test fluid. Figure 2(B-i) shows the captured microscopic images for representing the variations in the interface with respect to the flow rate of the test fluid (*Q_t_*) (*Q_t_* = 0.5, 2, and 4 mL/h). The corresponding interfaces of each flow rate were obtained as *β* = 0.3 ± 0.007 (*Q_t_* = 0.5 mL/h), 0.605 ± 0.004 (*Q_t_* = 2 mL/h), and 0.748 ± 0.004 (*Q_t_* = 4 mL/h). Using Equation (3), *C_P_* was then calculated at various flow rates of the test fluid. Figure 2(B-ii) shows the *C_P_* from the numerical simulation (□) and experiments (○) with respect to *β*. The *C_P_* tended to decrease when the interface was located near the left wall. At *β* < 0.3, there was a significant deviation between the simulated and experimental results. However, the *C_P_* increased gradually at *β* > 0.4. There was a slight difference between these results. Here, based on the variations in *C_P_* obtained from the experimental results, a linear regression technique (EXCEL^TM^, Microsoft, Redmond, WA, USA) was conducted to obtain the polynomial expression for *C_P_* as a function of *β*. The linear regression analysis gave a correction factor as *C_p_* = −9.014*β*^4^ + 21.273*β*^3^ − 18.403*β*^2^ + 7.051*β* − 0.168 (*R*^2^ = 0.99). By substituting the polynomial expression of *C_P_* into Equation (3), the analytical formula of the viscosity of test fluid is given as:

(4) $$\mu_{t}=\mu_{r}\times \left(\frac{\beta }{1-\beta }\right)\times \left(\frac{Q_{r}}{Q_{t}}\right)\times C_{P}\;\left(\beta \right)$$

The blood viscosity was then obtained by substituting the interface (*β*) into Equation (4).

### 3.2. Contributions of RBC Sedimentation for Control Blood

In previous studies, a driving blood syringe was set in the vertical direction (i.e., upright or inverted). In addition, the hematocrit was set to relatively lower levels than the normal level of 45% (i.e., 0.1% [17], 20% [11], 25% [30], and 30% [21,31]). The hematocrit of the blood tended to increase over time when the blood was supplied from the bottom region of the syringe into the microfluidic device. However, in this study, the blood syringe was set in the horizontal direction. It was expected that the RBC sedimentation might exhibit different patterns over time because of the different installation direction of the blood syringe. The hematocrit of the blood tended to decrease continuously over time after the blood entered the microfluidic channel. Here, instead of RBC aggregation-enhanced blood, control blood was tested to evaluate the contributions of hematocrit to blood flow under RBC sedimentation of the control blood in the blood syringe. The hematocrit of the control blood was adjusted to Hct = 20%, 25%, 30%, and 40% by adding normal RBCs into autologous plasma. The contributions of the hematocrits were evaluated experimentally by changing the hematocrit level, in which the diluent was fixed to the same plasma.

**Figure 3:**
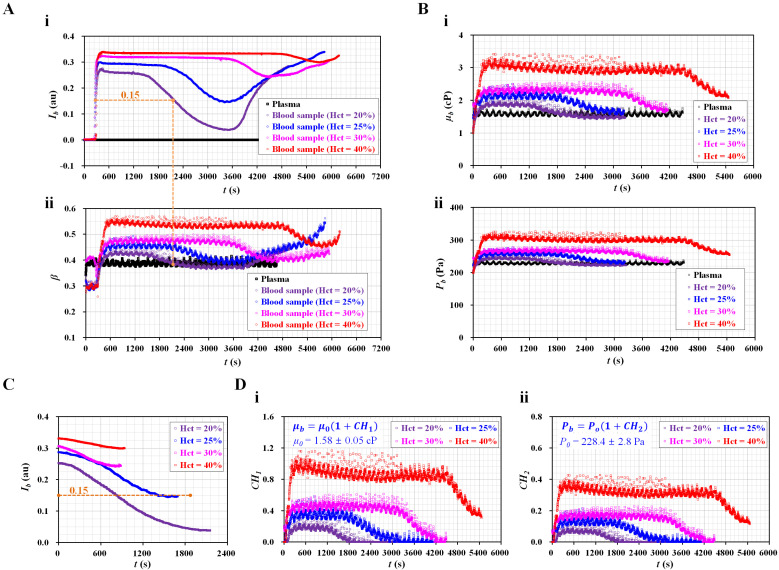
Quantitative evaluations of RBC sedimentation for the control blood. The hematocrit was adjusted to Hct = 20%, 25%, 30%, and 40% by adding normal RBCs into autologous plasma. (**A**) Temporal variations in *I_b_* and *β* with respect to Hct. (**A**-**i**) Temporal variations in *I_b_* with respect to Hct. (**A**-**ii**) Temporal variations in *β* with respect to Hct. (**B**) Temporal variations in *µ_b_* and *P_b_* with respect to Hct. (**B**-**i**) Temporal variations in *µ_b_* with respect to Hct. (**B**-**ii**) Temporal variations in *P_b_* with respect to Hct. (**C**) Temporal variations in *I_b_* with respect to hematocrit. Here, the *I_b_* was recollected from the time it began to decrease from flat value. (**D**) The contribution of RBC sedimentation to blood biophysical properties (blood viscosity and junction pressure). Two equations (*µ_b_* = *µ*_0_ [1 + *CH*_1_] and *P_b_* = *P*_0_ [1 + *CH*_2_]) were used to separate the contributions of RBCs and diluent in terms of blood viscosity and blood junction pressure. (**D**-**i**) Temporal variations in *CH*_1_ with respect to Hct. Here, *µ*_0_ = 1.58 ± 0.05 cP. (**D**-**ii**) Temporal variations in *CH*_2_ with respect to Hct. Here, *P*_0_ = 228.4 ± 2.8 Pa.

Figure 3(A-i) shows the temporal variations in *I_b_* with respect to Hct. Consequently, *I_b_* remained for a certain duration after the blood entered the blood channel. The value of flat *I_b_* increased substantially at higher levels of Hct ranging from 20% to 30%. A smaller difference was observed between Hct = 30% and Hct = 40%. The period of flat *I_b_* increased gradually with increasing Hct ranging from 20% to 40%. As shown in Figure 1(A-iii), RBC sedimentation caused a decrease in *I_b_* because the hematocrit decreased over time. Pure plasma had a zero value of *I_b_*. When the minimum value of *I_b_* was greater than zero, RBCs still flowed into the blood channel, even for blood (Hct = 20%). The minimum value of *I_b_* tended to increase at higher hematocrit values. After the diluent flowed into the blood channel for a certain duration, the RBCs entered the channel again. This caused *I_b_* to increase gradually over time. Figure 3(A-ii) shows the temporal variations in *β* with respect to Hct. The interface (*β*) exhibited trends similar to those of *I_b_*. Specifically, *β* tended to increase at higher levels of hematocrit. The period of flat *β* increased at a higher hematocrit. Interestingly, the two blood types (i.e., Hct = 20% and 25%) had minimum β similar to that of pure plasma (i.e., RBC-free diluent). As shown in Figure 3(A-i), when *I_b_* decreased to less than 0.15, the RBCs did not contribute to varying the *β*. Namely, small populations of RBCs (i.e., extremely lower hematocrit) did not have an influence on the interface in the coflowing channel. For that reason, when small RBCs or RBC-free diluent came into the coflowing channel, the *β* decreased to the same value of β for pure plasma. However, when the blood syringe was filled with blood with higher levels of hematocrit, ESR occurred over time. Diluent was not separated completely from blood during the ESR in the syringe. Thus, large populations of RBCs came into the microfluidic device. The minimum *β* had higher than that of plasma. Although the minimum value of *I_b_* increased significantly at higher Hct values, *β* did not exhibit a substantial difference (i.e., *I_b_* < 0.15). Based on the threshold of image intensity (i.e., *I_b_* = 0.15), it was possible to separate the contributions of RBCs (*I_b_* > 0.15) and diluents (*I_b_* < 0.15) by analyzing the temporal variations in *β*.

Figure 3(B-i) shows the temporal variations in blood viscosity (*µ_b_*) with respect to Hct. As expected, *µ_b_* increased significantly at higher levels of Hct. After the period of constant blood viscosity, RBC sedimentation caused a decrease in *µ_b_* over time. After an elapse of a certain time, the diluent was removed from the blood. Then, the diluents of the two blood samples (Hct = 20%, 25%) flowed into the coflowing channel. The viscosity of the diluent (i.e., plasma) exhibited similar values for two bloods: *µ*_0_ = 1.58 ± 0.05 cP. By substituting the temporal variations in *β* (Figure 3(A-ii)) into Equation (1), the blood junction pressure was then obtained with respect to the hematocrit. Figure 3(B-ii) shows the temporal variations in blood junction pressure (*P_b_*) with respect to Hct. The *P_b_* gradually increased at a higher level of hematocrit. RBC sedimentation resulted in decreasing *P_b_* significantly. When the two blood diluents (Hct = 20% and 25%) flowed into the coflowing channel, both diluents (i.e., plasma) had the same junction pressure of *P*_0_ = 228.4 ± 2.8 Pa. From these results, the contributions of the RBCs and diluent were determined, especially for the control blood with low hematocrit (Hct = 20% or 25%). In other words, it would be necessary for the control blood to be adjusted to a low hematocrit to obtain viscosity or junction pressure for the diluent and RBCs.

Based on conventional quantification [18], *I_b_* was recollected from the time at which it began to decrease to the time when it tended to increase again. Figure 3C shows the temporal variations in *I_b_* with respect to the hematocrit. As expected, the blood with a low hematocrit contributed significantly to decreasing *I_b_* over time. RBC sedimentation decreased with increasing Hct, ranging from 20% to 40%. However, blood flow (*I_b_* < 0.15) did not affect the contribution of RBCs to the mechanical properties (i.e., viscosity, junction pressure). When *I_b_* was greater than 0.15, the two blood samples (Hct = 20%, 25%) exhibited similar trends in *I_b_*. For the remaining two blood samples (Hct = 30%, 40%), the variation in *I_b_* decreased at a higher level of hematocrit. Thus, to consider the contribution of RBCs in the blood in terms of *I_b_*, it is necessary to select suitable data for quantifying RBC sedimentation. However, as shown in Figure 3(A-i), before *I_b_* began to decrease, it remained unchanged over time (i.e., *I_b_* > 0.15). The period of flat image intensity (i.e., *T_ESR_*) tended to increase with increasing hematocrit [32]. Thus, it would be better to quantify RBC sedimentation compared with conventional quantification (*I_ESR_*) [32]. As a limitation, *I_b_* did not provide enough information on the hematocrit from a mechanical point of view.

To quantify RBC sedimentation in the blood syringe, blood viscosity and junction pressure were obtained by analyzing the interface in the coflowing channel. Based on Einstein’s empirical expression for rigid spheres (i.e., *µ* = *µ_f_* [1 + 2.5 Φ]; *µ_f_*: the viscosity of the suspending fluid, and Φ: the volume fraction) [33], two formulas (i.e., *µ_b_* = *µ*_0_ [1 + *CH*_1_] and *P_b_* = *P*_0_ [1 + *CH*_2_]) were suggested to evaluate the contributions of hematocrit and diluent in terms of blood viscosity and blood junction pressure. Here, the subscripts *b* and *o* denote blood and plasma (or RBC-free diluent), respectively. Namely, *µ*_0_ and *P*_0_ denoted viscosity and pressure of the diluent, respectively. The effect of the hematocrit on blood flow was evaluated using two coefficients (*CH*_1_ and *CH*_2_). Here, the image intensity of the blood flow should be greater than 0.15. Otherwise, for *I_b_* < 0.15, *CH*_1_ = 0 and *CH*_2_ = 0. Figure 3(D-i) shows the temporal variations in *CH*_1_ with respect to Hct. As a result, the four types of blood had significantly different trends for *CH*_1_ when compared with *I_b_* (Figure 3C). In addition, *CH*_1_ tended to increase at a higher level of Hct. Figure 3(D-ii) shows the temporal variations in *CH*_2_ with respect to Hct. The *CH*_2_ exhibited similar variations in *CH*_1_. In conclusion, the two coefficients (*CH*_1_, *CH*_2_) provided quantitative information on RBC sedimentation in the blood syringe in terms of blood viscosity and blood junction pressure.

### 3.3. Contributions of RBC Sedimentation for Maximum RBC-Aggregated Blood

To accelerate RBC sedimentation in the blood syringe, the dextran solution was added into normal blood. Based on previous studies on physiological and pathological conditions [2,34], the maximum concentration of dextran solution was set at 20 mg/mL. To determine the contribution of hematocrit, the hematocrit was adjusted to Hct = 30%, 40%, and 50% by adding normal RBCs to the allowable concentration of the dextran solution.

**Figure 4:**
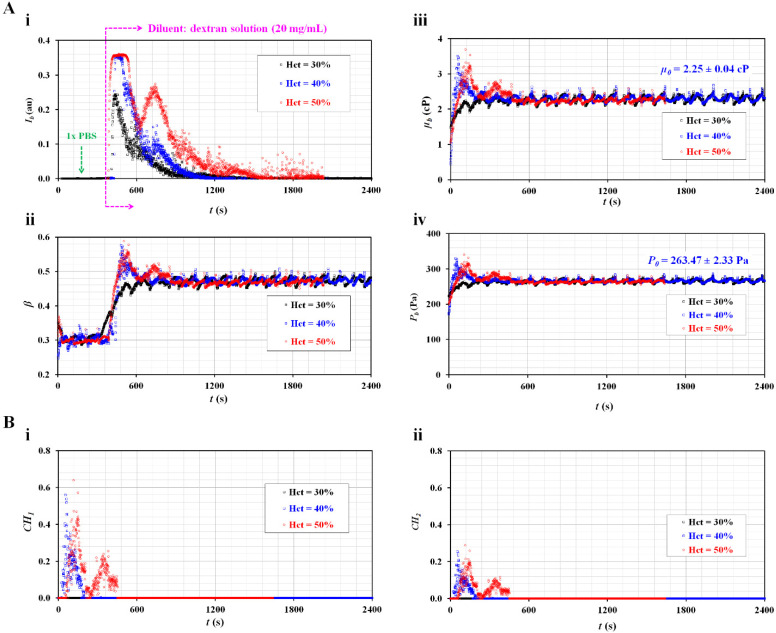
Quantitative evaluations of the effect of RBC sedimentation for maximum RBC aggregation-enhanced blood. The hematocrit was set at Hct = 30%, 40%, and 50% by adding normal RBCs into a dextran solution (20 mg/mL). (**A**) Temporal variations in image intensity and interface with respect to hematocrit. (**A**-**i**) Temporal variations in *I_b_* with respect to Hct. (**A**-**ii**) Temporal variations in *β* with respect to Hct. (**A**-**iii**) Temporal variations in *µ_b_* with respect to Hct. The viscosity of the diluent was *µ*_0_ = 2.25 ± 0.04 cP. (**A**-**iv**) Temporal variations in *P_b_* with respect to Hct. The junction pressure of the diluent was *P*_0_ = 263.47 ± 2.33 Pa. (**B**) The contributions of RBC sedimentation to the physical properties of blood (blood viscosity and junction pressure). (**B**-**i**) Temporal variations in *CH*_1_ with respect to Hct. (**B**-**ii**) Temporal variations in *CH*_2_ with respect to Hct.

Figure 4(A-i) shows the temporal variations in *I_b_* with respect to Hct. *I_b_* increased substantially when the blood flowed into the blood channel. Thereafter, RBC sedimentation significantly caused a decrease in *I_b_* over time. When compared with the control blood (Figure 3(A-i)), the dextran solution caused a considerable decrease in the duration of flat image intensity. The *I_b_* of the blood (Hct = 30%) decreased substantially over time without a flat image intensity. That is, the *I_b_* decreased substantially at lower hematocrits. Figure 4(A-ii) shows the temporal variations in *β* with respect to Hct. The *β* of the blood (Hct = 30%) reached a steady value without a peak. Blood RBCs did not have an influence on the blood flow. However, the remaining two blood samples (Hct = 40% and 50%) had peak values of *β*. RBCs in the blood caused an increase in *β*. However, the *β* reached a steady value early. Figure 4(A-iii) shows the temporal variations in *µ_b_* with respect to Hct. Within a specific time, when the image intensity was less than 0.15, the viscosity of the diluent was *µ*_0_ = 2.25 ± 0.04 cP.

As shown in Figure 4(A-ii), the blood viscosity increased for a short period for the two blood samples (Hct = 40%, 50%). The viscosity of blood (Hct = 30%) was the same as that of the diluent (C_dex_ = 20 mg/mL). Figure 4(A-iv) shows the temporal variations in *P_b_* with respect to Hct. The junction pressure of the diluent was determined to be *P*_0_ = 263.47 ± 2.33 Pa. The trends of *P_b_* were similar to those of *µ_b_*.

Based on the temporal variations in *µ_b_* and *P_b_* as shown in Figure 4(A-iii, A-iv), the two coefficients (*CH*_1_ and *CH*_2_) were obtained over time. Figure 4(B-i) shows the temporal variations in *CH*_1_ with respect to Hct. *CH*_1_ remained zero for the blood (Hct = 30%). However, the *CH*_1_ of the blood (Hct = 50%) was higher than that of the blood (Hct = 40%). Figure 4(B-ii) shows the temporal variations in *CH*_2_ with respect to Hct. Consequently, the variation in *CH*_2_ was similar to that of *CH*_1_. The results indicate that blood (Hct = 40% or 50%) exhibited the effects of RBC sedimentation when RBCs were added into a specific concentration of dextran solution (20 mg/mL). However, blood (Hct = 30%) was not found to be effective for detecting the contributions of RBC sedimentation in blood syringes. In the succeeding experiments, the hematocrit was set to 50% by adding RBCs into dextran solutions of various concentrations.

### 3.4. Quantitative Comparison between the Proposed and Conventional Methods for RBC Aggregation-Enhanced Blood

Based on the quantitative results as discussed above, the present method was applied to measure the contributions of RBC sedimentation for various concentrations of dextran solution, which has been commonly used to enhance RBC sedimentation or RBC aggregation in blood. By referring to previous studies [2,34], five different kinds of dextran solution were prepared by setting the concentration of dextran solution to C_dex_ = 5, 7.5, 10, 15, and 20 mg/mL. Based on contributions of hematocrit to ESR, as shown in Figure 4, the hematocrit of each blood was adjusted to 50% by adding normal RBCs to individual dextran solutions. The flow rates of the two fluids were set to 0.5 mL/h.

**Figure 5:**
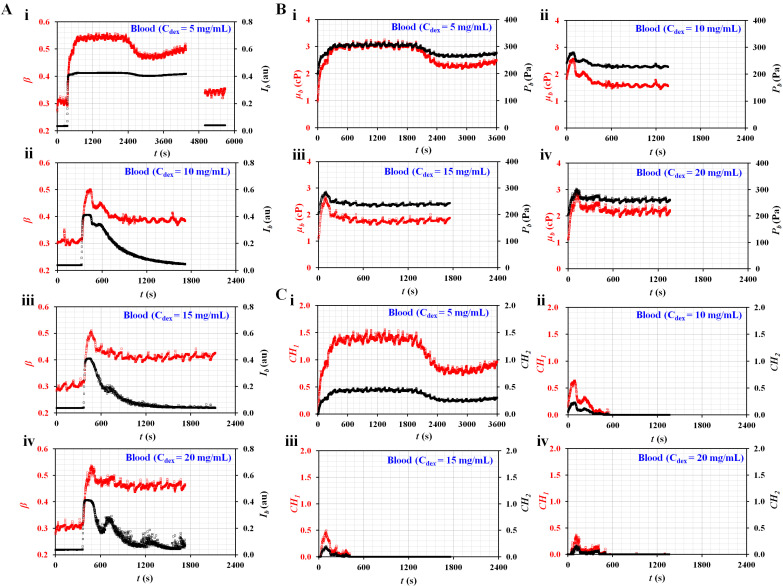
Quantitative evaluations of RBC sedimentation for RBC aggregation-enhanced bloods. The degree in RBC sedimentation was controlled by preparing dextran solution with various concentrations. The hematocrit was set at Hct = 50%. The flow rates of both fluids were set at 0.5 mL/h. (**A**) Temporal variations in *I_b_* and *β* with respect to the concentration of the dextran solution (C_dex_) ((**A**-i) C_dex_ = 5 mg/mL, (A-ii) C_dex_ = 10 mg/mL, (A-iii) C_dex_ = 15 mg/mL, and (A-iv) C_dex_ = 20 mg/mL). (**B**) Temporal variations in *µ_b_* and *P_b_* with respect to C_dex_ ((B-i) C_dex_ = 5 mg/mL, (B-ii) C_dex_ = 10 mg/mL, (B-iii) C_dex_ = 15 mg/mL, and (B-iv) C_dex_ = 20 mg/mL). (**C**) Temporal variations in *CH*_1_ and *CH*_2_ with respect to C_dex_ ((C-i) C_dex_ = 5 mg/mL, (C-ii) C_dex_ = 10 mg/mL, (C-iii) C_dex_ = 15 mg/mL, and (C-iv) C_de*x*_ = 20 mg/mL).

Figure 5A shows the temporal variations in *I_b_* and *β* with respect to the concentrations of dextran solution (C_dex_) ((i) C_dex_ = 5 mg/mL, (ii) C_dex_ = 10 mg/mL, (iii) C_dex_ = 15 mg/mL, and (iv) C_dex_ = 20 mg/mL). Here, the *I_b_* of the blood (C_dex_ = 5 mg/mL) did not decrease below 0.15. Thus, after the experiment, a dextran solution without RBCs (C_dex_ = 5 mg/mL) was tested to obtain *I_b_* and *β* over time. Variations in *β* and *I_b_* were added after *t* = 4800 s. It was then possible to obtain *µ*_0_ and *P*_0_ of the diluent (i.e., C_dex_ = 5 mg/mL). However, the remaining blood samples were separated into blood and diluent. After an elapse of a certain time, the *I_b_* of each blood decreased to below 0.15. The *µ*_0_ and *P*_0_ of each blood were obtained by analyzing the temporal variations of *β*, which remained unchanged over time. Figure 5B shows the temporal variations in *µ_b_* and *P_b_* with respect to C_dex_ ((i) C_dex_ = 5 mg/mL, (ii) C_dex_ = 10 mg/mL, (iii) C_dex_ = 15 mg/mL, and (iv) C_dex_ = 20 mg/mL). Except for the blood (C_dex_ = 5 mg/mL), the dextran solution contributed to accelerating RBC sedimentation substantially. The viscosity and junction pressure had an early peak value. They remained unchanged for a long time. Figure 5C shows the temporal variations in *CH*_1_ and *CH*_2_ with respect to C_dex_ ((i) C_dex_ = 5 mg/mL, (ii) C_dex_ = 10 mg/mL, (iii) C_dex_ = 15 mg/mL, and (iv) C_dex_ = 20 mg/mL). As a result, the blood (C_dex_ = 5 mg/mL) had higher values of *CH*_1_ and *CH*_2_ for a longer period than the other bloods. The dextran solution contributed to accelerating RBC sedimentation in the blood syringe. Furthermore, the two coefficients (*CH*_1_ and *CH*_2_) calculated from viscosity and junction pressure exhibited distinctive trends with respect to the concentration of the dextran solution.

**Figure 6:**
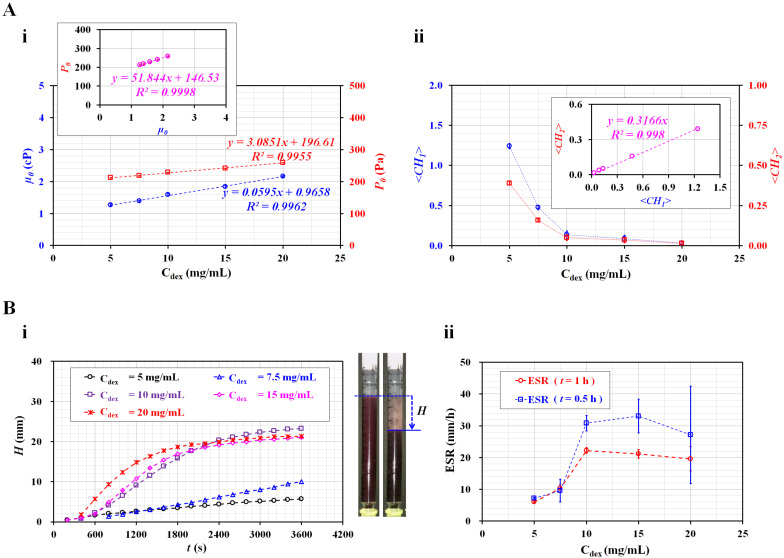
Quantitative comparison between the present and conventional ESR methods for RBC aggregation-enhanced bloods. (**A**) Variations in four factors (i.e., *µ*_0_, *P*_0_, <*CH*_1_>, and <*CH*_2_>) obtained using the present method. (**A**-**i**) Variations in viscosity (*µ*_0_) and junction pressure (*P*_0_) of the diluent with respect to C_dex_. (**A**-**ii**) Variations in <*CH*_1_> and <*CH*_2_> with respect to C_dex_. (**B**) Quantitative measurement of ESR using a conventional ESR method. (**B**-**i**) Temporal variations in RBC sedimentation (*H*) with respect to C_dex_. Right side panel showed snapshots of the RBC sedimentation of a blood sample (C_dex_ = 20 mg/mL) at *t* = 0 and 1 h. (**B**-**ii**) Variations in ESR obtained at *t* = 0.5 h and 1 h with respect to C_dex_.

Figure 6A summarizes the variations in four parameters (i.e., *µ*_0_, *P*_0_, <*CH*_1_>, and <*CH*_2_>) with respect to C_dex_. Here, <*CH*_1_> and <*CH*_2_> denote the arithmetic averages of *CH*_1_ and *CH*_2_ obtained for *t* = 1500 s (i.e., *n* = 1500). Figure 6(A-i) shows the variations in *µ*_0_ and *P*_0_ of the diluent with respect to C_dex_. Viscosity increased linearly with respect to C_dex_ (*µ*_0_ = 0.0595 C_dex_ + 0.9658, *R*^2^ = 0.9962). In addition, the junction pressure increased linearly with respect to C_dex_ (*P*_0_ = 3.0851 C_dex_ + 196.61, *R*^2^ = 0.9955). The inset shows a scatter plot for evaluating the linear relationship between *P*_0_ and *µ*_0_. As the coefficient of linear regression had a higher value of *R*^2^ = 0.9998, either *P*_0_ or *µ*_0_ can be used effectively to detect differences in the diluent (i.e., concentration of dextran solution). Figure 6(A-ii) shows the variations in <*CH*_1_> and <*CH*_2_> with respect to C_dex_. Both coefficients exhibited consistent variations with respect to C_dex_. They decreased substantially between C_dex_ = 5 and 10 mg/mL. For blood with a higher concentration of C_dex_ = 10 mg/mL, <*CH*_1_> and <*CH*_2_> decreased gradually with respect to C_dex_. The inset shows a scatter plot for validating the linear relationship between <*CH*_1_> and <*CH*_2_>. As the coefficient of linear regression had a relatively high value of *R*^2^ = 0.998, either <*CH*_1_> or <*CH*_2_> can be used to effectively monitor variations in RBC sedimentation in blood syringes.

For comparison with the results obtained using the present method, the ESR was measured with a conventional ESR method. In the modified ESR method, a 1 mL disposable syringe was filled with 1 mL blood, and installed vertically against the gravitational direction. Figure 6(B-i) shows the temporal variations in RBC sedimentation (*H*) with respect to C_dex_. The *H* tended to increase gradually for three bloods (C_dex_ = 5, 7.5, and 10 mg/mL). However, for bloods that were higher than C_dex_ = 10 mg/mL, the *H* was saturated above 2400 s, approximately. Subsequently, substantial RBC sedimentation did not occur over time.

The right panel of Figure 6(B-i) shows snapshots of RBC sedimentation of blood (C_dex_ = 20 mg/mL) at *t* = 0 and 1 h. As the RBC sedimentation saturated before 1 h, two specific periods for calculating the ESR (i.e., *t* = 0.5 h and 1 h) were selected instead of *t* = 1 h. Using the conventional ESR method, the ESR was calculated at a falling velocity (mm/h) by measuring the RBC sedimentation during two specific durations (*t*) (*t* = 0.5 h, 1 h). Figure 6(B-ii) shows variations in ESR estimated at *t* = 0.5 h and 1 h with respect to C_dex_. For the ESR obtained at *t* = 1 h, the dextran solution (C_dex_ ≤ 10 mg/mL) contributed to increasing the RBC sedimentation in the blood syringe. For an amount higher than C_dex_ = 10 mg/mL, the ESR decreased slightly with the increasing dextran solution concentration. As the RBC sedimentation saturated before 1 h, the scattering (or standard deviation) of the ESR was relatively lower. However, when the ESR was quantified at 0.5 h, the ESR showed large fluctuations above C_dex_ = 10 mg/mL. Compared with previous studies [11,20,31], the results showed consistent trends with respect to the concentration of the dextran solution. Here, as normal RBCs were added to the dextran solution, the diluent (i.e., dextran solution) caused the ESR to increase. In fact, the conventional ESR did not separate the contributions of RBCs and diluent in the blood. Additionally, the conventional ESR was obtained by monitoring the interface between RBCs and diluent in the vertical tube. When compared with the conventional ESR method, the present method provided quantitative information on diluent as well as blood, such as viscosity and junction pressure. In addition, as blood viscosity and blood junction pressure were strongly related to hematocrit, the two coefficients obtained from the two formulas (i.e., *µ_b_ = µ*_0_ [1 + *CH*_1_], *P_b_ = P*_0_ [1 + *CH*_2_]) could quantify variations in RBC sedimentation in the blood syringe. Thus, it was possible to determine why the blood had a higher ESR level. As a distinctive advantage, the present method can separate the contribution of RBC and diluent in the blood.

As a limitation of this study, the present method was demonstrated using suspended bloods (i.e., healthy RBCs in dextran solution or plasma for validating proof of concept). It was not a generalized study with various bloods collected from patients. In the near future, it will be necessary to validate the performance of the present method with clinical disease bloods [2,13,35,36]. Furthermore, to improve the portability of the present experimental setup, the two syringe pumps will be replaced by portable micropumps [37,38,39].

## 4. Conclusions

In this study, by analyzing the blood flow in microfluidic channels, viscosity and junction pressure were employed to quantify RBC sedimentation in a blood syringe. During blood delivery from the blood syringe to the microfluidic device, the diluent was separated from the blood after an elapse of a certain time. The blood and diluent (i.e., RBC-free fluid) were then sequentially supplied to a microfluidic device. To quantify the contributions of RBCs or diluents in terms of viscosity and junction pressure, image intensity and interface were obtained by supplying blood and reference fluid into the blood channel and coflowing channel. Based on the threshold intensity of blood flow image (i.e., diluent for *I_b_* < 0.15, RBCs for *I_b_* > 0.15), two coefficients (*CH*_1_, *CH*_2_) obtained from the empirical formulas (i.e., *µ_b_ = µ*_0_ [1 + *CH*_1_], *P_b_ = P*_0_ [1 + *CH*_2_]), which were used to quantify the contributions of RBCs (hematocrit) or RBC sedimentation. The present method was applied to obtain the viscosity and junction pressure for various suspended bloods (i.e., healthy RBCs in dextran solution or plasma). Based on experimental investigations, four parameters (i.e., *µ*_0_, *P*_0_, *CH*_1_, and *CH*_2_) were considered effective for quantifying the contributions of RBCs (or hematocrit) and diluent. In particular, when quantifying the contributions of RBC sedimentation, the two coefficients (*CH*_1_ and *CH*_2_) had more consistent trends than the conventional ESR method. In conclusion, it was found that the present method can provide biomechanical information on blood in terms of viscosity and junction pressure. Additionally, it can be employed to effectively detect RBC sedimentation in a blood syringe.
